# Pennsylvania

**Published:** 1897-02

**Authors:** 


### LEGISLATION.

*Pennsylvania.*—At the present legislative session a bill has been introduced looking to the registration, examination, and licensing of horseshoers. A feature of the Act is the compulsory registration of all master and journeymen shoers. Registration is based upon a license from a board of examiners. All apprentices, journeymen, or master horseshoers for a period of four years will, upon proper affidavit being presented, be exempt from examination and allowed to register. The board of examiners is to consist of one veterinarian, two master horseshoers, and two journeymen shoers; they are to be appointed by the Governor for a period of one, two, three, four, and five years. The registration fee is to be two dollars. The board is to elect its own officers, hold suitable meetings, pay all expenses from fees received, and allow only one officer, the secretary, a salary of five hundred dollars. The board shall maintain for public inspection a master and journeymen horseshoers' register. The Act provides penalties for violation of its provisions; defines apprentices, journeymen, and master shoers, and makes provision for an annual report to the Governor. This bill will be strongly urged by the various State and local organizations, has the support of the national organization, and bids fair to become a law.

Governor Hastings, in his address to the Legislature, has this to say of State work: "The State Veterinarian, under the direction of the Live-Stock Sanitary Board, has been actively engaged in suppressing outbreaks of contagious diseases among farm animals. A large number of diseases of domestic animals has been dealt with, and in some instances serious loss has been prevented by prompt and well-directed measures. More attention has been given to tuberculosis than to any other single disease, because many cattle-owners have applied for assistance in freeing the herds of this scourge. Coöperation between the farmers and this Board has resulted in the eradication of tuberculosis in many districts. Serious hindrance to the eradication of many destructive diseases is found to result in not making prompt report to the Live-Stock Sanitary Board. It is the duty of the State Veterinarian to investigate the cause of every epidemic affecting the lives and health of domestic animals. When we consider that $125,000,000 is invested in live-stock in this Commonwealth, and that the yearly loss resulting from diseases that may be prevented is estimated at $6,000,000, the importance of closer relations between the farmer and the Live-Stock Sanitary Board becomes apparent."

The ordinance prepared for the consideration of Council in the city of Pittsburg, looking to the most salutary provisions for guarding the milk-supply and regulating the sale thereof, has been withdrawn on second reading, on advice of the City Solicitor that part of the same was unconstitutional. The provisions in question have just been declared constitutional by the Supreme Court of Minnesota.

